# Prognostic factors in transnasal endoscopic surgery for paediatric patients with ossifying fibroma of the paranasal sinuses and skull base

**DOI:** 10.1186/s40463-023-00641-w

**Published:** 2023-07-26

**Authors:** Jingying Ma, Bing Zhou, Qian Huang, Shunjiu Cui, Dingfang Cao

**Affiliations:** 1 Department of Otolaryngology–Head and Neck Surgery, Beijing Tongren Hospital, Capital Medical University, Key Laboratory of Otolaryngology and Head and Neck Surgery, Ministry of Education, 1 Dong Jiao Min Xiang, Dongcheng District, Beijing, 100730 People’s Republic of China; 2 Department of Pathology, Beijing Tongren Hospital, Capital Medical University, Beijing, People’s Republic of China

**Keywords:** Ossifying fibroma, Recurrence, Transnasal endoscopic surgical resection, Paediatric

## Abstract

**Background:**

Ossifying fibroma of the paranasal sinuses and skull base in paediatric patients is difficult to operate and can recur easily after surgery. This study aimed to analyse factors associated with recurrence after transnasal endoscopic resection of ossifying fibroma in paediatric patients.

**Methods:**

This retrospective observational study included 34 patients under 17 years of age who underwent transnasal endoscopic resection of ossifying fibroma of the paranasal sinuses and skull base from 2005 to 2021 at a single tertiary medical centre. Clinical indicators such as age; surgical history; pathological type; intraoperative bleeding; and orbit, anterior skull base, sphenoid bone, sella turcica, clivus, or frontal sinus involvement were subjected to univariate analysis using the χ^2^ test, to investigate whether any of these factors affected recurrence.

**Results:**

All 34 patients underwent transnasal endoscopic resection. The follow-up period was 6–120 months (mean: 48.0 months). Five patients experienced local recurrence during the follow-up period (14.7%). Results of χ^2^ tests indicated that a history of previous surgery, the amount of intraoperative bleeding, and sphenoid and/or sella turcica and clivus involvement were significantly associated with recurrence (*P* < 0.05). Age; pathological stage; and orbit, anterior skull base, and frontal sinus involvement were not associated with recurrence (*P* > 0.05).

**Conclusions:**

The increased risk of recurrence after transnasal endoscopic resection of nasal–skull base ossifying fibroma should be considered during endoscopic surgery in paediatric patients with a history of previous surgery, intraoperative bleeding tendency, and sphenoid and/or sella turcica and clivus involvement. These patients require careful postoperative follow-up.

## Introduction

Ossifying fibroma (OF) is a benign fibrous bone lesion defined by the World Health Organization (WHO) as a well-demarcated lesion composed of fibrocellular tissue and mineralised material of varying appearances. OF is classified into conventional OF and juvenile OF (JOF) [1]. Conventional OF occurs primarily in adults [2]. JOF occurs in children or adolescents and has an aggressive growth pattern, with destruction of cortical bone or involvement of the nasal cavity, orbit, or even the interior of the skull. In 2005, the WHO indicated the age of onset of JOF as under 15 years [1]; however, JOF also occurs in adults [3, 4]. Based on its histopathological features, JOF is classified into juvenile psammomatoid ossifying fibroma (JPOF) or juvenile trabecular ossifying fibroma (JTOF) subtypes [1].

Transnasal endoscopic surgical resection is the primary treatment for nasal–skull base OF. Because of its location, the tumour has a rich blood supply, increasing intraoperative bleeding risk and hampering accurate tumour boundary determination intraoperatively. Furthermore, children’s narrow nasal cavities make it challenging to achieve complete tumour resection.

Here, we report on the perioperative and follow-up condition of 34 patients under 17 years of age who underwent transnasal endoscopic resection of nasal–skull base OF at our institute from 2005 to 2021, and analyse possible factors associated with recurrence.

## Methods

### Patient characteristics

Charts of thirty-four patients (23 male, 11 female; age range: 1 year, 10 months to 17 years; mean age: 12.1 years) under 17 years of age, with nasal sinus OFs that were resected via transnasal endoscopic surgery under imaging navigation between May 2005 and December 2021 at the Department of Otolaryngology Head and Neck Surgery in Beijing Tongren Hospital, were reviewed. Diagnosis was confirmed by medical history, nasal endoscopy, imaging, and pathological examination.

### Preoperative nasal sinus computed tomography and imaging preparation

All patients underwent preoperative sinus computed tomography (CT) using a Sytec 4000*i* whole-body CT scanner (GE, Healthcare, Chicago, IL, USA). The slice thickness was 0.67–1 mm. Continuous scanning was performed, without spacing. CT scan data were imported into BrainLab (Munich, Germany) or Medtronic Fusion (Minneapolis, MN, USA) workstations for 3D reconstruction and image processing.

### Surgical procedure

**Fig. 1:**
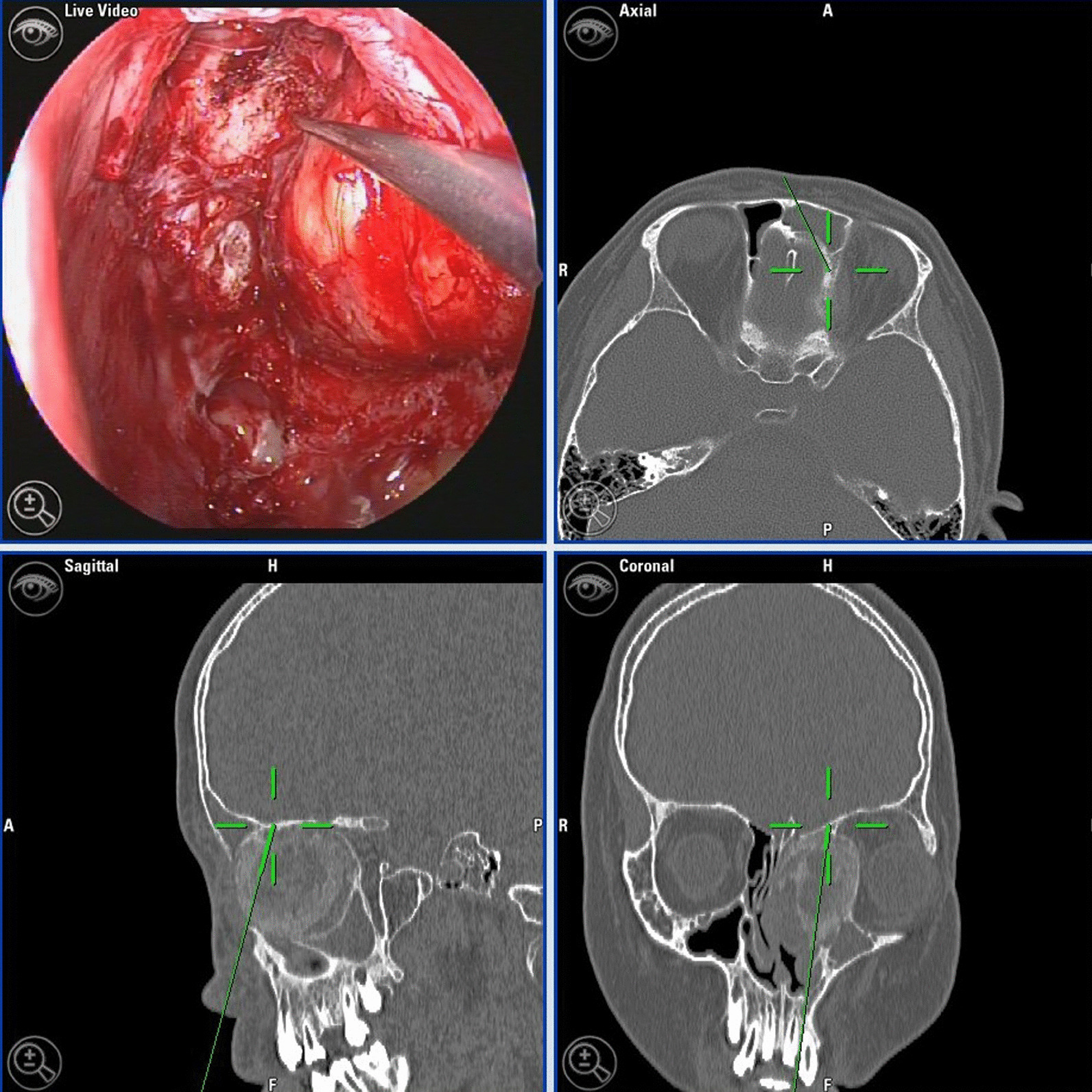
Screenshot of image navigation-guided transnasal endoscopy during the procedure. The probe indicates the skull base site, and the navigation 3D image indicates that the probe is located near the skull base

In all patients, intravenous general anaesthesia and intraoperative controlled hypotension were used, and transnasal endoscopic surgery was performed under imaging navigation. Intraoperatively, the mass was highly haemorrhagic, and we tried to swiftly resect abnormal tissue using Takahashi rongeurs or a curette to reach its outer rim as quickly as possible. The haemorrhage stopped, and the surgical field gradually improved when the bulk of mass was almost completely removed. Then, a diamond bone drill was used to grind down the tumour cementum (Fig. 1). The usual procedural choice was to detach all the pathological tissue of the anterior skull base or orbital wall until the healthy dura or orbital fascia was exposed, using this as an anatomical reference for complete resection of the tumour along its margins. If the optic nerve canal was involved, then the tumour was marked by the orbital fascia, the affected bone was removed using a diamond drill, the optic nerve sheath was exposed by following the orbital apex to the optic nerve in the anterior to posterior direction, and a budesonide cotton dressing was used to relieve swelling and provide protection. For areas where the bony covering could not be removed, a diamond drill was used to grind the bone until the cortex was smooth. In cases of frontal sinus and frontal recess involvement, the tumour was exposed and removed using the Draf IIb or Draf III procedure. For tumours wrapping around the anterior ethmoid artery in the frontal recess, the tumour was located intraoperatively under image navigation and was coagulated. Intraoperative monopolar or bipolar electrocoagulation of the supplying vessels was used in conjunction with haemostatic dressings, SURGICEL Absorbable Hemostat (Ethicon Inc., Raritan, NJ, USA), and other materials for haemostasis.

### Postoperative nasal sinus CT re-examination

At 3–5 days after resection of the nasal mass, postoperative nasal sinus CT re-examination (Sytec 4000*i* whole-body CT scanner; GE; 2-mm slice thickness; 5-mm layer spacing) was performed to establish a follow-up observation baseline.

### Follow-up

For postoperative follow-up, transnasal endoscopic follow-up management and annual nasal sinus CT was performed. Recurrence was defined as confirmed tumour recurrence on CT imaging.

### Variables analysed

Based on the literature and clinical experience, the following factors were selected as variables for analysis that may be associated with recurrence: age; history of surgery (previous transnasal endoscopic or open OF resection at another hospital); pathological type; intraoperative blood loss; orbit involvement; anterior skull base involvement; sphenoid, sella turcica, and clivus involvement; and frontal sinus involvement.

### Statistical analysis

Univariate χ^2^ testing was performed (SPSS v26; IBM SPSS Inc., Armonk, NY, USA) to determine associations of variables with tumour recurrence, and *P* < 0.05 was determined to be statistically significant.

## Results

**Fig. 2:**
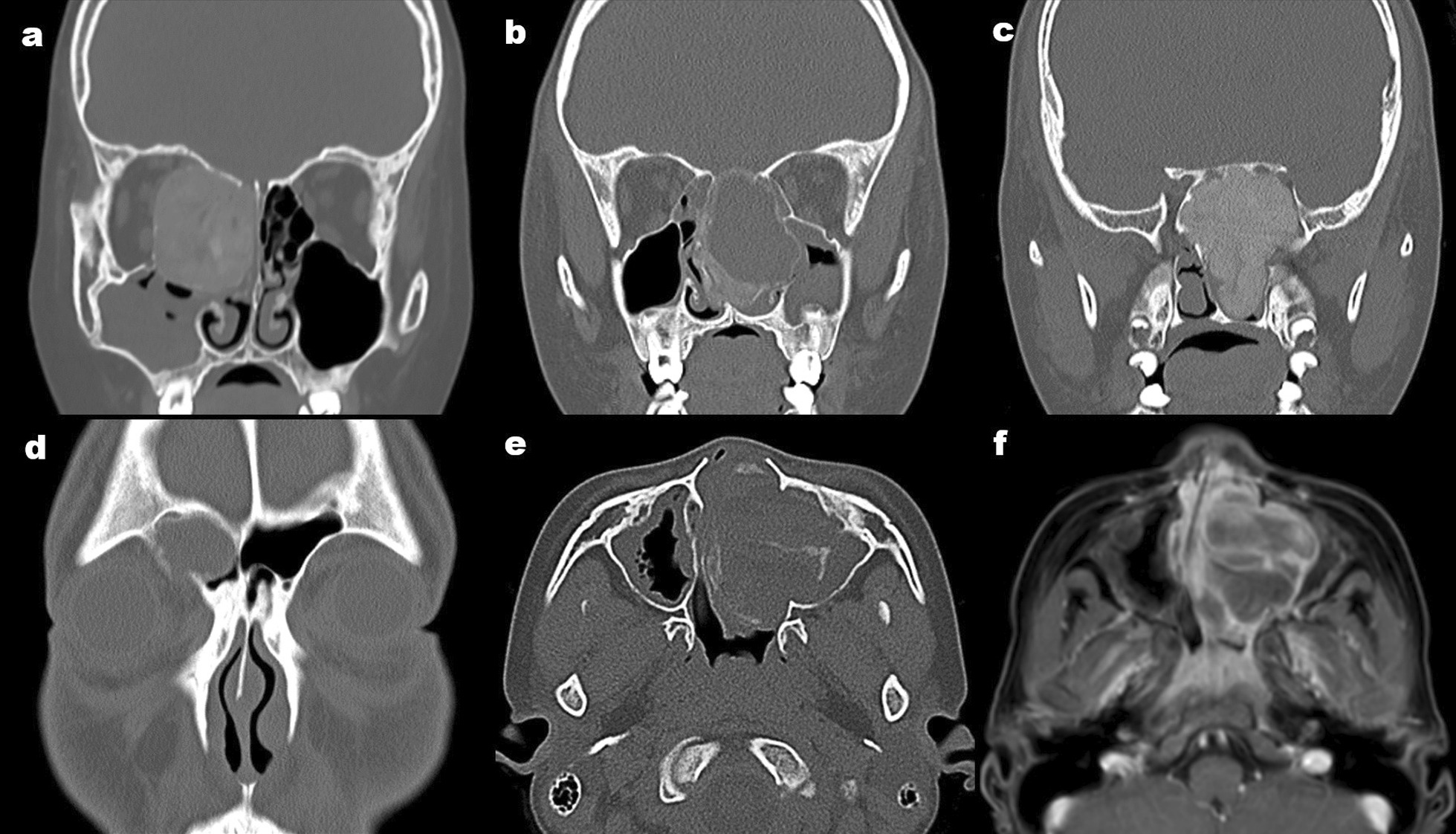
Preoperative coronal computed tomography of the nasal sinus (**a**–**d**), preoperative image of juvenile ossifying fibroma (OF) with an aneurysmal bone cyst (**e**), and enhanced magnetic resonance imaging of an aneurysmal bone cyst (**f**) in children with OF. **a** Tumour with orbit involvement, **b** anterior skull base involvement, **c** tumour with sphenoid and sella turcica involvement, **d** tumour with right frontal sinus involvement, **e** multifocal cystic lesion with peripheral bone shell formation, **f** more obvious enhancement of the cyst wall and intracapsular septum of the lesion

The disease course ranged from 4 months to 5 years. Twenty-six patients underwent a first surgery, and eight patients had a history of previous surgery. All patients presented with a single, round, space-occupying mass in the nasal sinus, involving a large, glassy, opaque tumour with soft tissue density, of varying size, and with intact cementum around the tumour on nasal sinus CT, with expansive growth, and distorted but clearly demarcated surrounding tissues (Fig. 2). Of the 34 patients, 30 patients demonstrated orbital wall (Fig. 2a), 24 patients had anterior skull base (Fig. 2b), eight patients had sphenoid and/or sella turcica and clivus (Fig. 2c), and five patients had frontal sinus (Fig. 2d) involvement. There were two cases of secondary aneurysmal bone cyst (ABC), which presented as low-density bone swelling with single or multiple compartments, a round shape, a thin bone shell at the margins, and a fluid–fluid interface on CT (Fig. 2e). The latter was clearly visible on MRI, with a hyperintense signal above the fluid interface on T1-weighted imaging (T1WI) and T2WI, and an isointense to hypointense signal below the interface. The cystic wall and intracapsular lesion septum were generally enhanced clearly on enhanced scans, while the intracystic part was not enhanced (Fig. 2f).

The surgical duration was 90–540 min, with an average of 252 min. The intraoperative blood loss was 50–4000 mL, with an average of 1076 mL. Twelve patients had blood loss ≥ 1000 mL and 22 patients had blood loss < 1000 mL (Table 1). There were four patients with periorbital ecchymosis after surgery, which gradually relieved in about 2 weeks. There were no other complications.

**Table 1:** Factors analysed for postoperative recurrence of paediatric ossifying fibroma

Variables	Number of patients	Number of recurrences (%)	*P* value
Age			0.840
< 15 years	26	4 (15.4)	
≥ 15 years	8	1 (12.5)	
History of surgery			0.037
Yes	8	3 (37.5)	
No	26	2 (7.7)	
Pathological type			0.146
JPOF	25	5 (20.0)	
JTOF	9	0 (0)	
Intraoperative blood loss			0.024
≥ 1000 ml	12	4 (33.3)	
< 1000 ml	22	1 (4.5)	
Orbit involvement			0.536
Yes	30	4 (10.0)	
No	4	1 (25.0)	
Anterior skull base involvement			0.574
Yes	24	3 (12.5)	
No	10	2 (20.0)	
Sphenoid, sella turcica, and clivus involvement			0.037
Yes	8	3 (37.5)	
No	26	2 (7.7)	
Frontal sinus involvement			0.084
Yes	5	2 (40.0)	
No	29	3 (10.3)	

**Fig. 3:**
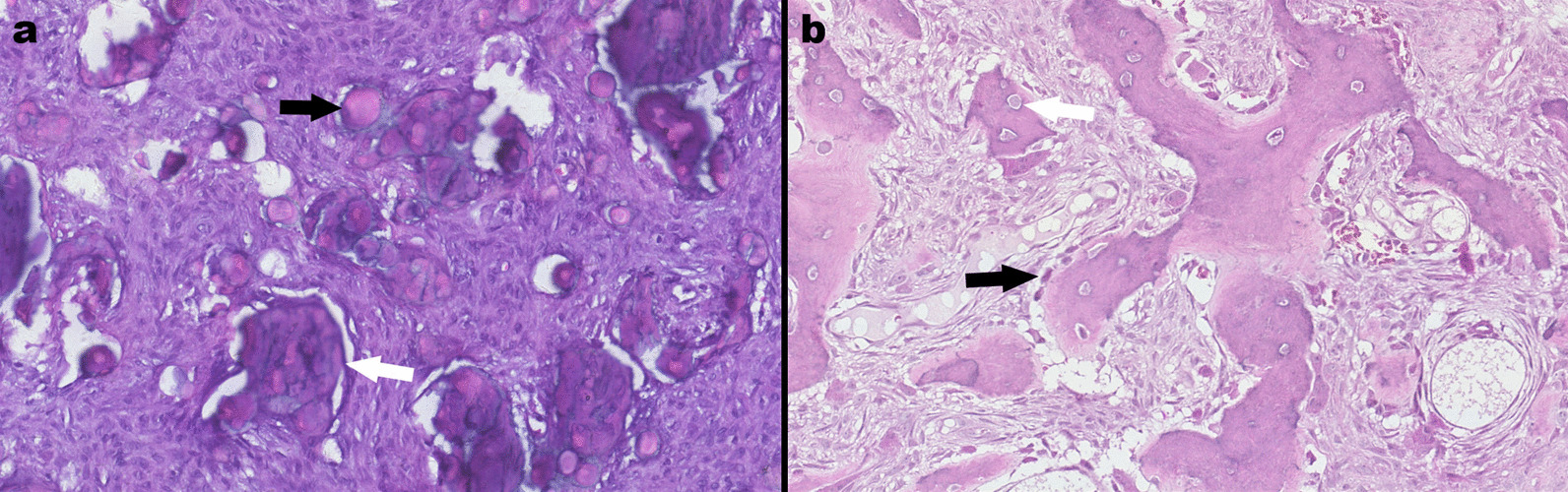
Juvenile psammomatoid ossifying fibroma (OF) (**a**) and juvenile trabecular OF (**b**). **a** Black arrow, concentrically lamellated particles; white arrow, irregular thorn-like calcified strands (haematoxylin and eosin staining; ×40); **b** white arrow, immature bone trabeculae containing irregular osteoblasts; black arrows, lining of encapsulated enlarged osteoblasts (haematoxylin and eosin staining; ×40)

All patients had JOF, which included 25 cases of JPOF and nine cases of JTOF. Histologically, JPOF showed diffusely distributed ‘cementum-like’, irregularly shaped bone deposits in the tumour tissue, showing eosinophilic or deeply basophilic staining and containing varying numbers of osteocytes, surrounded by eosinophilic collagenous material. A few basophilic, concentrically lamellated particles were seen in the tumour tissue. Osteocytes were absent from the tumour bodies (Fig. 3a). JTOF showed trabeculae of immature bone that anastomosed to form a lattice containing irregularly shaped osteocytes surrounded by hypertrophic osteoblasts (Fig. 3b).

**Fig. 4:**
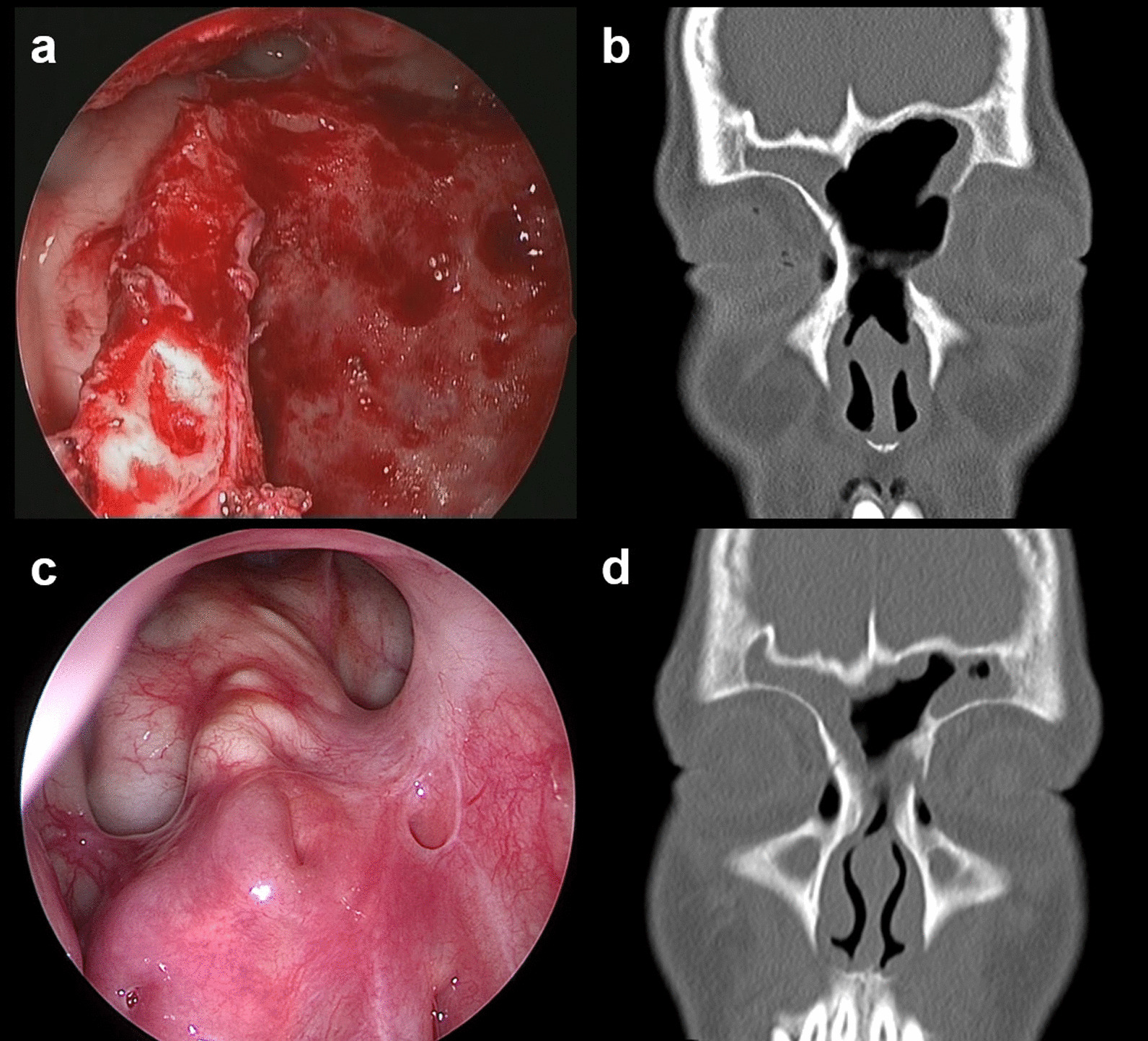
Postoperative follow-up images of ossifying fibroma of the frontal sinus. **a** Endoscopic images after complete tumour resection via the extended Draf IIb procedure, **b** postoperative nasal sinus coronal and axial computed tomography (CT) scans indicating complete tumour resection, **c** 4-year postoperative follow-up endoscopic images, **d** 4-year postoperative nasal sinus CT re-examination indicating no tumour recurrence

All postoperative follow-up nasal sinus CT examinations indicated complete tumour resection. Follow-up included transnasal endoscopic and nasal sinus CT re-examination (Fig. 4). The follow-up period ranged from 6 to 120 months with an average of 48.0 months. Five cases of local recurrence were found on follow-up CT (14.7%). Four patients required repeat operation, including one 11-year-old child who was followed up until the age of 18 years, at which time transnasal endoscopic resection was performed again. The remaining patient was followed up for 3 years, and surgery was not indicated at that time.

A history of previous surgery (37.5% vs. 7.7%, *P* = 0.037), intraoperative blood loss exceeding 1000 mL (33.3% vs. 4.5%, *P* = 0.024), and sphenoid and/or sella turcica and clivus involvement (37.5% vs. 7.7%, *P* = 0.037) were significantly associated with OF recurrence (*P* < 0.05). Age; pathological type; and orbit, anterior skull base, or frontal sinus involvement were not associated with recurrence (*P* > 0.05).

## Discussion

In this study, we showed that a history of previous surgery, the intraoperative blood loss amount, and sphenoid and/or sella turcica and clivus involvement were significantly associated with recurrence in paediatric patients with nasal–skull base OF (*P* < 0.05). We found no statistical evidence for association of age; pathological type; or orbit, anterior skull base, or frontal sinus involvement with recurrence (*P* > 0.05); however, this does not necessarily indicate that these factors are not involved in OF surgery outcomes.

Complete resection is the first-line treatment for OF. The aggressiveness of tumour growth and susceptibility to surrounding structures, such as the anterior skull base and orbit, often lead to complicated surgery and recurrence due to incomplete tumour removal. Open surgical procedures, such as transcranial, transorbital, and transfacial approaches, may require craniofacial and orbital reconstruction [5]. Postoperative OF recurrence rates are reportedly 12–28% [2, 6]. The transnasal endoscopic approach preserves the integrity of craniofacial bones and structures and is now the mainstay of OF treatment. Using endoscopic surgery can reduce the incidence of OF recurrence [7, 8]. In the present study, the recurrence rate of OF after transnasal endoscopic surgery was 14.7%. Since nasal endoscopic surgery requires intra-tumoral operation, finding and locating the tumour margin is essential for complete tumour resection. We found an association between a history of previous surgery and recurrence (*P* < 0.05), which suggests that failure of complete resection in prior surgery, or recurrent tumour growth, with unclear tumour shell margins relative to the primary tumour, increases the difficulty of re-operation and hampers complete resection. Charnovic et al.[9] and Liu et al.[10] reported that recurrence rates between tumours with clear and unclear margins on CT imaging differed by about 25%. The use of intraoperative image navigation probe guidance can help determine tumour invasion sites and tumour margins, which facilitate complete resection and reduce recurrence.

The intraoperative blood loss amount was ≥ 1000 mL in 12 patients (four with recurrence), while blood loss was < 1000 mL in 22 patients (one with recurrence). The intraoperative blood loss amount was significantly associated with tumour recurrence (*P* < 0.05). This suggests that the rich blood supply of OF and associated excessive blood loss pose a risk during resection. A large intraoperative blood loss results in an unclear visual field, which affects intraoperative judgment of the tumour margins, impacting complete resection. One case of recurrence in the group with less intraoperative blood loss was a patient aged 1 year and 9 months. Although only about 500 mL of intraoperative blood loss occurred, it was relatively large given that the child weighed about 12 kg and had a blood volume of about 1000 mL (about 8% of body weight). Thus, the blood loss was relatively high, increasing circulatory failure risk, which impacted the surgical resection efficacy. Therefore, controlling and reducing intraoperative blood loss is crucial. In addition to controlling hypotension, to ensure circulatory stability, the tumour should be resected as soon as possible during operation. When only the ‘bony shell’ remains after tumour resection, bleeding is significantly reduced. An image navigation probe can be used to assist in determining the tumour margins when excessive bleeding occurs. Haemostatic materials, such as haemostatic dressings, SURGICEL Absorbable Hemostat, and epinephrine gauze, can be used for haemostasis.

We found no statistically significant association of orbit, anterior skull base, or frontal sinus involvement with tumour recurrence. Complete ‘en bloc’ OF resection, particularly the bony shell tissue, is essential for preventing tumour recurrence. After most of the tumour is resected, the tumour margins are determined using an image navigation probe, and the bony shell is ground thin using a diamond bone drill and then detached to resect the lesion completely. Most of the tumours in this group involved the orbit (30/34, 88.2%) and anterior skull base (24/34, 70.6%). The dura mater and orbital fascia at the anterior skull base are the key intraoperative anatomical reference markers that guide complete resection of the tumour shell. The dura mater or orbital fascia is easily revealed first. The tumour margins are clearly defined using this marker, after which the tumour and the bony shell are carefully detached using microinstruments. For tumours that invade the frontal recess and frontal sinus, extended Draf IIb or Draf III surgery can be used to expose the tumour margins fully and achieve complete tumour resection. In the present study, there were four cases of orbital fascia injury, and periorbital ecchymosis occurred after the surgery. It is suggested that protection of the integrity of the orbital fascia and dura during surgery is one of the key techniques, and it is also important to prevent complications.

There were three cases of recurrence (3/8) where the tumour did, and two cases (2/26) where it did not, involve the sella turcica and clivus (*P* < 0.05). Thus, sphenoid and/or sella turcica and clivus involvement is associated with recurrence. This may be because tumours involving the sphenoid and sella turcica invade major skull base structures, such as the optic canal, superior orbital fissure, cavernous sinus, and internal carotid artery, and tumour dissection carries major risks, particularly in patients with normal vision. Hence, it is necessary to locate the tumour margins, internal carotid artery, and optic nerve canal under image navigation guidance and determine the extent of resectable tumour, based on the three-dimensional anatomical structural relationships. Tumours involving the greater sphenoid bone wing, the clivus, and the occipital bone have a deeper location, and complete removal of the tumour bony shell is more difficult. A diamond grinding drill can be used to grind the bone cortex to a thin and smooth surface. Surgical safety is the primary goal, and complete removal of the bony shell is not necessary. Residual tumour shell tissue and adjacent bone structure should be re-examined by nasal sinus CT immediately post-surgery to establish a follow-up baseline, after which regular follow-up CT examinations should be performed to facilitate early recurrence detection, monitoring, and treatment.

The OF pathological type has also been reported to influence recurrence rate. Although JOF mostly occurs in children and adolescents, it has a wide age range (3 months to 72 years) [3]. The JTOF subtype recurs more frequently (36.8%) than does the JPOF subtype (33.3%) [6]. JTOF is regarded as a highly aggressive disease with frequent recurrence [9]. Our 34 cases were selected from paediatric and adolescent patients under 18 years of age, and all cases were JOF, including 25 JPOF cases (5 recurrent cases) and nine JTOF cases (no recurrence). Pathological subtype and recurrence were not associated. This result may be due to the different histopathological characteristics of JPOF and JTOF, affecting different age groups and having different tendencies to occur at different sites.JPOF has a broader age range and occurs primarily in the frontal nasal–orbital–ethmoid complex, whereas JTOF has a younger age range, is more aggressive, and primarily involves the mandible, with only a slight tendency for maxilla involvement [9, 11]. In our patients, OF occurred primarily in the skull base, nasal sinus, and nasal cavity, and our study included relatively few cases of JTOF.

Previous studies have shown that aggressive growth and recurrence are more common in younger age groups [3, 12]. However, Charcanovic et al. [9] reported no statistically significant correlation between age and recurrence. JPOF is reported more frequently in adults than is JTOF, and it shows a wider age distribution than does JTOF, which is predominantly seen in younger patients. It is considered that these lesions may persist for a long time from puberty onward, although they manifest in adulthood only when they reach a significant size. Therefore, they are still classified as juvenile lesions [3]. In the present study, all patients were under 18 years of age, and no significant correlation between age and recurrence was found, which may be because all cases in this group were JOF, with more cases of the JPOF subtype. However, the age distribution suggests that patients younger than 14 years may have a higher tendency to relapse after surgery. The postoperative images indicate that this finding may be related to the fact that younger children were compromised by their anatomical environment and could not undergo as larger resection as adults could. In other words, complete removal of the bony shell is the basic strategy to prevent recurrence.

Two of our cases included secondary aneurysmal bone cysts, which are expansile, multilocular, osteolytic lesions containing multinucleated giant cells and fibrous septa with compartmentalised haemorrhage of the surrounding reactive bone. Approximately half of nasal sinus ABCs are secondary to a fibro-osseous lesion, of which OF is the most common, with approximately 3.4% OF cases associated with nasal sinus ABC [13]. Whether ABC alters OF prognosis is unclear, given the rarity of these cases in the literature [14–16]. Currently, endoscopic surgery is recommended to treat OF in nasal sinus ABC, due to its minimal invasiveness. However, early detection and complete surgical resection is necessary due to the locally invasive nature and high recurrence rate (28.57%) of these lesions [13].

Long-term follow-up with nasal endoscopy and nasal sinus CT is essential for detecting tumour recurrence and preventing possible complications. Treatment of recurrence must be considered based on a combination of factors, such as age, pathological type, and recurrence site. When the recurrent tumour is small and no serious symptoms such as vision loss affect function, continuous follow-up observation may be considered; otherwise, re-operation may be needed. The timing of re-operation also requires consideration of all these factors. One 11-year-old patient with JPOF in this study developed recurrence in the frontal sinus region and was followed up until age 18 years, at which point the patient underwent transnasal endoscopic Draf III surgery, after which no recurrence occurred over a 6-year follow-up period. In another 11-year-old child with JPOF, local recurrence occurred in the maxillary sinus wall at 6 months post-surgery. With regular CT re-examination, we observed that the disease developed slowly during a 3-year follow-up. There was no obvious functional impact, and no revision surgery has been performed to date.

## Conclusions

In summary, our preliminary results suggest that a history of previous surgery, a large amount of intraoperative blood loss, and involvement of the sphenoid and/or sella turcica and clivus increase recurrence risk of nasal–skull base OF after transnasal endoscopic resection in paediatric patients. This should be considered during endoscopic surgery and postoperative follow-up. Adequate haemostasis is needed during surgery. Imaging navigation can help determine tumour margins to facilitate complete tumour removal and minimise recurrence. Long-term, close postoperative follow-up is needed to monitor recurrence and to select the appropriate surgical procedure and timing.

## Data Availability

Available on request.
